# Gait-to-Gait Emotional Human–Robot Interaction Utilizing Trajectories-Aware and Skeleton-Graph-Aware Spatial–Temporal Transformer

**DOI:** 10.3390/s25030734

**Published:** 2025-01-25

**Authors:** Chenghao Li, Kah Phooi Seng, Li-Minn Ang

**Affiliations:** 1 School of Internet of Things, Xi’an Jiaotong-Liverpool University, Taicang 215000, China; chenghao.li23@student.xjtlu.edu.cn; 2 School of Science, Technology and Engineering, University of the Sunshine Coast, Petrie, QLD 4502, Australia; lang@usc.edu.au

**Keywords:** social robot, human–robot interaction, emotion classification

## Abstract

The emotional response of robotics is crucial for promoting the socially intelligent level of human–robot interaction (HRI). The development of machine learning has extensively stimulated research on emotional recognition for robots. Our research focuses on emotional gaits, a type of simple modality that stores a series of joint coordinates and is easy for humanoid robots to execute. However, a limited amount of research investigates emotional HRI systems based on gaits, indicating an existing gap in human emotion gait recognition and robotic emotional gait response. To address this challenge, we propose a Gait-to-Gait Emotional HRI system, emphasizing the development of an innovative emotion classification model. In our system, the humanoid robot NAO can recognize emotions from human gaits through our Trajectories-Aware and Skeleton-Graph-Aware Spatial–Temporal Transformer (TS-ST) and respond with pre-set emotional gaits that reflect the same emotion as the human presented. Our TS-ST outperforms the current state-of-the-art human-gait emotion recognition model applied to robots on the Emotion-Gait dataset.

## 1. Introduction

**Figure 1:**
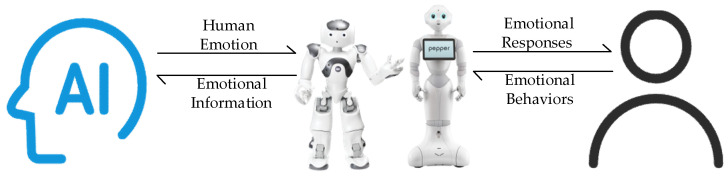
An emotional human–robot interaction system between robotics and humans supported by machine learning methods.

Recent advancements in neural networks have sparked exploration across various modalities, enabling more efficient feature extraction in fields such as Natural Language Processing (NLP), Computer Vision (CV), and Healthcare Diagnostics. Human–Robot Interaction (HRI) is a multifaceted research area that encompasses a wide range of information, including audio, visual, text, and signal, with a growing emphasis on applying machine learning methods to enhance the intelligence of these systems. A critical factor in improving HRI intelligence is granting social capabilities to the robots [1], which classifies them as social robots. Social robots are designed to actively engage with humans to achieve their internal social aims [2], necessitating accurate recognition of human behaviours. To achieve this, a fundamental requirement is the robot’s ability to understand, reason about human emotions [3], and present emotional responses [4]. Recent studies have highlighted that robots equipped with emotional HRI system (Figure 1) capabilities can create a positive and sociable impression on humans [5]. The integration of Large Language Models (LLMs) in robots has significantly advanced the development of humanized communication within emotional HRI [6]. For instance, the framework proposed by the authors in [7] introduces a multimodal emotional HRI system, which combines visual and auditory modalities to enhance the quality of human–robot companionship. In this system, the robot conveys emotions through facial expressions and language generated by GPT models. Furthermore, emotional HRI has been shown to play a central role in mental health care. Studies [8,9] indicate that interactions between humans and robots, such as storytelling and conversational robots based on LLMs, can provide valuable support for individuals in expressing their emotions, particularly those who face difficulties with emotional articulation.

A key challenge in achieving emotional interactions between humans and robots lies in accurately recognizing human emotions. Convolutional neural networks (CNNs) are widely used for inferring human emotions based on facial expressions, and this methodology has become prevalent in the development of emotional HRI [7,10,11,12]. Bath et al. [12] demonstrated that CNNs with residual blocks can effectively recognize emotions from facial expressions. Additionally, Yu et al. [13] incorporated Long Short-Term Memory (LSTM) networks into CNNs to further enhance the performance of emotion recognition systems. However, facial emotion recognition faces significant challenges under varying lighting conditions, head orientations, and facial occlusions [14]. Additionally, processing facial images is computationally expensive, as the required resolution for accurate recognition demands large image sizes [15]. While these issues can be mitigated by imposing constraints on human behaviour or controlling the ambient environment, such approaches compromise the sociability of the robot and the naturalness of HRI. To overcome these limitations, alternative affective modalities are necessary, with human gait emerging as a potential solution.

Gaits are composed of a series of skeleton graphs that record the coordinates of human joints during walking. Before the emergence of emotional gaits analysis, the research mainly focused on recognizing human emotion through facial expressions, speech intonation, and physiological signals [16], as gaits lack both efficient affective feature learning methods and large-scale datasets. While gaits have not been widely studied in emotion recognition, it has played a crucial role in human action recognition, where the initial challenge of extracting representative features from gaits has been addressed. Influenced by the development of CNNs and graph convolutional networks (GCNs), Yan et al. [17] introduced a model capable of capturing both temporal patterns and spatial connections between joints in gait graphs. Inspired by this novel approach to affective learning, Bhattacharya et al. [18] released a large-scale emotional gait dataset, containing 2177 real gaits labelled with emotional information. This work addressed two major challenges in gait-based emotion recognition: effective feature extraction and dataset availability. As a result, various machine learning techniques have been developed, making gait a more popular affective modality. Current research has demonstrated that the utilization of gait offers advantages in terms of low acquisition cost [19,20] and the ability to be monitored from long distances [21]. Although some studies have explored emotional gait in robots [22,23], the integration of human emotional gait perception with robot emotional gait responses remains an underexplored area.

Despite the issues related to capturing affective information having been solved by the spatial–temporal graph convolutional network (ST-GCN) in [17,18], current spatial–temporal approaches for extracting representative affective information have two key limitations: ignoring sequential dependency and lacking structural understanding of the graph. Current ST-GCN based methods rely on CNNs to extract temporal information. While CNNs can effectively capture global representative features over specific periods, they fail to account for the sequential dependencies between elements in the data. This type of temporal relationship, where each element is dependent on previous elements, is more effectively captured by Recurrent Neural Networks (RNNs). However, RNNs suffer from inefficient training due to their inability to parallelize computations [24]. Another challenge arises from merely aggregating spatial information based on connections between nodes, which ignores the global position of nodes and the substructures within the graph. This can lead to nodes becoming indistinguishable after aggregation, thereby weakening the representational power of graph convolutional networks (GCNs) on the graph level [25]. These issues become more pronounced in graphs with complicated structures. As there is a trend toward enhancing the performance of ST-GCNs in emotion prediction by introducing additional connections in the gait graphs, such as reconstructing them into fully connected graphs [26], it is crucial to explore methods that strengthen the positional and structural representations in skeleton-based graphs.

To bridge the gap in emotional HRI research based on gait analysis, our study introduces a Gait-to-Gait Emotional HRI system. This system emphasizes human-gait emotion classification and the design of predefined robotic emotional gait responses. First, we capture videos of the walking person using the camera on the NAO robot and extract the human gaits. Our Trajectories- and Skeleton-Graph-Aware Spatial–Temporal Transformer (TS-ST) model then predicts the person’s emotion and transmits this predicted emotion to the NAO robot. Finally, the NAO expresses the same emotion through its preset gaits. Our TS-ST model is designed to address the challenges described above in extracting both temporal and spatial information. Inspired by the success of State Space Models (SSMs) in NLP [27], which excel in capturing sequential dependencies, we applied the SSM to encode the trajectorial information in the gaits’ sequences, incorporating temporal attention to mitigate the limitations in representing temporal data. Our spatial transformer utilizes the Laplacian and Random Walk encodings to enhance the extraction of spatial information by comprising node positions and graph substructures in the gait graphs.

In summary, we propose a new emotional HRI approach based on human gaits, introducing the Gait-to-Gait Emotional HRI system utilizing the NAO robot. To the best of our knowledge, we are among the first to integrate both human emotional gait and robot emotional gait in emotional HRI.

We present a novel spatial–temporal transformer-based model that extracts affective representations by considering both sequential dependencies in the frames and positional, as well as structural, information in the graph. Our approach incorporates State Space Models (SSM) and Graph Transformers into gait-based emotion recognition.

## 2. Related Works

### 2.1. Social Robots and Emotional Human–Robot Interaction

The growth of research on robotics and advancements in hardware have expanded the applications of robots beyond utilization in the industry, bringing them into everyday life as social robots. The exploration of the role of social robots in society has been ongoing, with particular emphasis on developing interactive methods for human–robot interaction. Rasouli et al. [28] demonstrated the potential of social robots in reducing anxiety during clinical interventions, while a personalised household robot for caring for the elderly was proposed by Di Napoli et al. [29]. Despite exploring the utility of social robots, another stream of research emphasizes improving the social intelligence of robots, where sharing emotions between humans and robots is a feasible method. Early research conducted by Niculescu et al. [30] showed that emotional expression by robots can increase the users’ positive feeling and their willingness to interact. However, limited by hardware constraints, their research only investigated the influence of a robot’s voice during speech. With advancements in machine learning and robotics, recent researchers have been able to design systems that include both human emotion recognition and robotic emotional responses. Park et al. [11] introduce an emotional interaction framework between humans and robots. They employ a trained CNN model to predict one of five human emotions, and their robot responds with the same emotion using animated facial expressions. A similar emotional HRI framework based on facial emotions is proposed by Bagheri et al. [31], where they utilize reinforcement learning for human emotion recognition. In a more recent study, Liu et al. [7] applied human speech to enhance the accuracy of facial emotion recognition in companion robots. While facial emotion recognition and response [10,32,33] have become a mainstream approach in emotional HRI, the potential of emotional gait in this domain remains largely unexplored.

### 2.2. Gait Emotion Recognition

Gaits are powerful modalities to express human emotions, while the utilization of deep learning approaches to extract affective representations starts from Long Short-Term Memory (LSTM) networks. Randhavane et al. [34] presented a vanilla LSTM network to recognize four emotions, happy, angry, sad, and neutral, from human gaits. However, the initial recognition accuracy of their LSTM model was limited, as LSTM networks only capture temporal information in gait sequences, lacking spatial context related to skeleton graphs. This gap in spatial information was soon addressed by Bhattacharya et al. [18]. Inspired by the success of ST-GCN [17] in gait-based action recognition, Bhattacharya et al. demonstrated that the representatively spatial features in emotional gaits can be obtained by the GCN, while CNNs replace the LSTM networks to capture temporal features. Their lightweight ST-GCN significantly improved emotion recognition accuracy compared to the prior LSTM-based method. However, the replacement of the LSTM networks neglects the inherent sequence dependencies among frames of gaits. Subsequently, numerous studies following this thought of incorporating CNNs and GCNs [35,36]. Hu et al. [37] identified a limitation in methods based on the combination of CNNs and GCNs: the inability to capture long-range relationships in the temporal and spatial domains of gait data. To address this, they proposed a transformer and CNN-based method to capture affective information in gaits, pioneering the use of transformers for gait-based emotion recognition. However, these methods still lacked comprehensive utilization of inherent information in gait graphs, such as the positions of nodes and the substructures of graphs. In terms of applying gait emotion analysis to robotics, Narayanan et al. [5] demonstrated a navigation approach for a social robot in a crowd-crossing scenario, based on the recognition of human-gait emotions. They utilized CNNs to extract affective representations, allowing the robot to plan navigation routes based on the predicted emotions of observed individuals. However, integrating human gait recognition with robotic emotional gaits as responses remains an underexplored aspect in the application of human emotional gait recognition with robots.

### 2.3. Graph Transformer

Transformers have achieved significant achievements across various areas, largely due to their global attention mechanism, which allows elements in sequences to attend to all others [38]. In contrast, Graph Convolutional Networks (GCNs) are limited in their ability to capture global or long-range information in graphs [39]. Consequently, a natural thought emerged in the field of graph learning: utilizing transformers to replace GCNs for acquiring long-range dependencies in graphs. Ying et al. [40] proposed a transformer-based graph learning model called Graphormer, highlighting the importance of encoding the structures of graphs. Their works demonstrate that Graphormer outperforms mainstream GCNs on graph-level prediction tasks on large-scale graphs. Although this emphasizes the potential advantages of transformers in graph learning with their ability to capture long-range dependencies, the short-range information within graphs is neglected. To further improve the performance of graph transformers in graph representation, research has focused on the incorporation of transformers and GCNs. Rampášek et al. [41] introduced a feasible framework for graph transformers that combines transformers and GCNs in parallel. They also proposed methods to fuse various positional and structural encodings, thereby enhancing the graph transformer’s ability to understand graph topology. The impressive performance of graph transformers has made them a research focus in applications such as protein engineering [42], drug-target affinity prediction [43], recommended systems [44], and action recognition [45]. However, graph learning methods for emotional gait analysis have yet to be explored extensively.

## 3. Methodology

**Figure 2:**
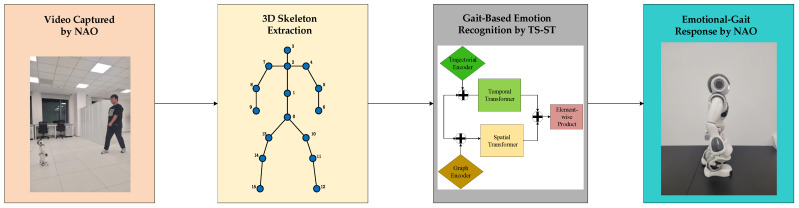
Overview of Gait-to-Gait Emotional HRI system.

We propose a new emotion-driven gait-based emotional HRI system, named the “Gait-to-Gait Emotional HRI system”, recognizing human emotions from gaits and responding with predefined emotional gaits using the NAO robot. An overview of the entire system is illustrated in Figure 2. Initially, human gaits are recorded using the camera on NAO’s head. A pretrained HoT model is then used to extract the 3D gait coordinates from the video data, with detailed information on the HoT model available in [46]. The extracted gait data are subsequently fed into our novel gait emotion classifier, TS-ST, to infer the emotion of the observed human. Finally, the NAO robot expresses the predicted emotion through its predefined gaits, enabling emotional HRI.

In the subsequent subsections, we describe our approaches for emotion classification and gait-based robotic response in detail. We first introduce the skeletal representations of the emotional gaits, followed by a detailed discussion of the architecture of our TS-ST model and its modules. Finally, we demonstrate how the NAO’s gaits are employed to express different emotions.

### 3.1. Definition of Gait Skeletal Graph

A sequence of gait graphs G consists of skeletal graphs G extracted from T frames in the video, defined as $G=\left(G^{1},G^{2},\ldots ,G^{T}\right)$, where the skeletal graph at frame t is defined as $G^{t}=(V^{t},E^{t})$, with t∈{1,2,…,T}. The set of nodes $V^{t}={\left\{v_{i}^{t}\right\}}_{i\in \{1,2,\ldots ,J\}}$ in the graph represents the joints, J, of the human skeleton. $E^{t}=\{e_{ij}^{t}=(v_{i}^{t},v_{j}^{t})|A_{ij}=1\}$ is the set of edges between node pairs, denoting the natural connections between joints in the human body. Here, $A_{ij}\in R^{J\times J}$ is the adjacency matrix of the skeletal graph, which indicates whether there is a connection between nodes $v_{i}^{t}$ and $v_{j}^{t}$. The definition of $A_{ij}$ can be written as follows:

(1) $$A_{ij}=\left\{\begin{array}{c} 1\;\;\;\;if\;there\;is\;connection\;between\;v_{i}^{t}\;and\;v_{j}^{t}\;\\ 0\;\;\;\;otherwise\;\;\;\;\;\;\;\;\;\;\;\;\;\;\;\;\;\;\;\;\;\;\;\;\;\;\;\;\;\;\;\;\;\;\;\;\;\;\;\;\;\;\;\;\;\;\;\;\;\;\;\;\;\;\;\;\;\;\;\;\;\;\;\; \end{array}\right.$$

**Figure 3:**
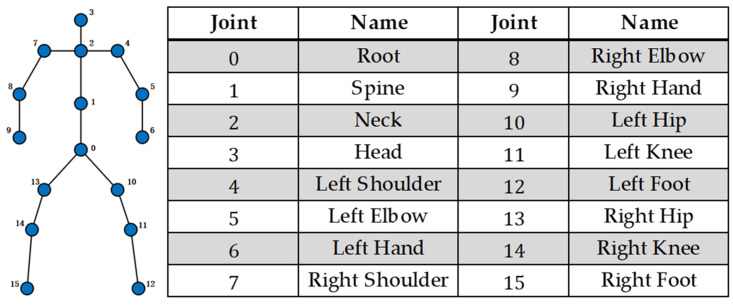
Illustration of skeletal graph.

The node features of the skeletal graph $G^{t}$ can be defined as $X^{t}={\left\{x_{i}^{t}\in R^{C}\right\}}_{i\in \{1,2,\ldots ,J\}}$, where $x_{i}$ coordinates of joint i in C dimensions. Figure 3 presents the structure of the gaits utilized for emotion recognition in this paper. The skeletal graph consists of 16 joints, each represented by three-dimensional coordinates that record their locations during walking. The sequence length for gait sequences is set to 240 frames, while the gait sequences shorter than this length are extended by repeating frames to match the required length.

### 3.2. Emotion Classifier Based on Gaits: TS-ST

**Figure 4:**
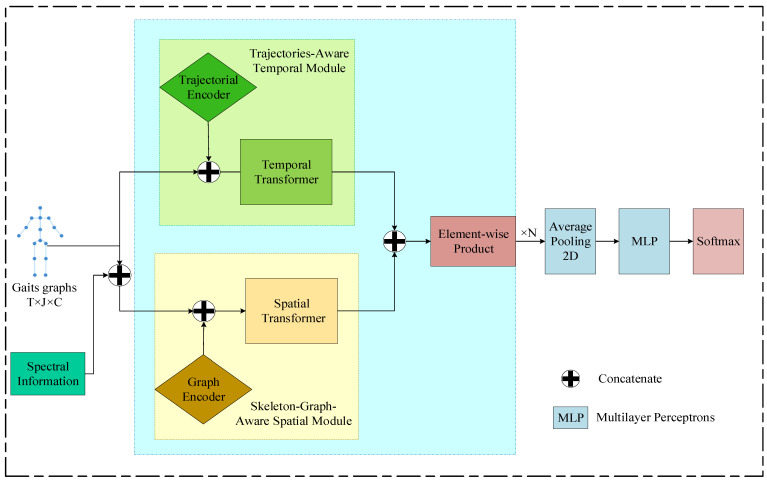
Overall architecture of Trajectories-Aware and Skeleton-Graph-Aware Spatial–Temporal Transformer (TS-ST).

The overall structure of our gait-based emotion classifier, TS-ST, is illustrated in Figure 4. The input gait graph sequence has the size of $R^{T\times J\times C}$, where T=240, J=16, and C=3. As described in the previous section, the input consists of T=240 frames, with each frame representing a gait graph containing J=16 joints. The position of each joint is defined by a three-dimensional coordinate (*x*, *y*, *z*), corresponding to *C* = 3 dimensions. Our TS-ST model is primarily composed of two parallel modules stacked in sequence: the Trajectories-Aware Temporal (TT) module and the Skeletal-Graph-Aware Spatial (SS) module. The TT module captures temporal representations by understanding sequence dependencies between frames, while the SS module extracts spatial representations by considering positional and structural information from the skeletal graph. The integration of these two modules, referred to as the TT-SS module, fuses the outputs of the TT and SS modules through an element-wise product operation. By stacking N TT-SS modules in sequence, the model generates affective representations that combine temporal and spatial information.

The resulting representations are averaged using 2D pooling across the T and J dimensions and are subsequently projected onto the emotion class space in the final dimension. Additionally, we incorporate spectral features of size $R^{J\times C}$ to enhance the classifier’s performance. These spectral features serve as complementary inputs, augmenting the global temporal information and manually derived affective features.

In the following subsections, we describe the methods for capturing temporal information with sequence dependencies and extracting positional and structural information from the skeletal graph, followed by the architectures and algorithms used in the TT and SS modules.

#### 3.2.1. State Space Models

State Space Models (SSMs) are extensively applied in estimating the output of a first-order differential system by mapping the sequence of continuous-time input 1-D signals $x(t)\in R^{L}$ to response signals $y(t)\in R^{L}$, which depend on the latent states $h(t)\in R^{N\times L}$ [27]. Equations (2) and (3) can be utilized to define SSMs, where $A_{SMM}\in R^{N\times N}$, $B_{SSM}\in R^{N\times 1}$ and $C_{SSM}\in R^{1\times N}$ are continuous parameters. In this context, L denotes the length of the input sequence, while N is determined by the predefined sizes of the parameters of $A_{SMM}$, $B_{SSM}$, and $C_{SSM}$. When processing an input sequence of length L with D channels, SSMs are applied to each channel independently. For example, in the case of the gait sequence described in Section 3.2, L corresponds to the number of frames, while D represents the number of dimensions in the joint coordinates.

(2) $$h\prime \left(t\right)=A_{SMM}h\left(t\right)+B_{SSM}x\left(t\right)$$

(3) $$y_{SSM}\left(t\right)=C_{SSM}h(t)$$

While h(t) is required to obtain the output sequence $y_{SSM}\left(t\right),$ computing h(t) in a continuous-time system is challenging. We can discrete the sequence $y_{SSM}\left(t\right)$, computing h(t) using a discretization step size Δ to address this. The resulting discrete ${y_{t}}^{SSM}$ and $h_{t}$ can be described by following Equations (4) and (5), which provides the output from the discrete system.

(4) $$h_{t}=\bar{A_{SMM}}h_{t-1}+\bar{B_{SSM}}x_{t}$$

(5) $${y_{t}}^{SSM}=\bar{C_{SSM}}h_{t}$$

The discrete parameters $\bar{A_{SMM}}$, $\bar{B_{SSM}}$, and $\bar{C_{SSM}}$ can be defined by following Equations (6)–(8).

(6) $$\bar{A_{SMM}}=exp(\Delta A_{SMM})$$

(7) $$\bar{B_{SSM}}={\left(\Delta A_{SMM}\right)}^{-1}(exp(\Delta A_{SMM}-I))\cdot \Delta B_{SSM}$$

(8) $$\bar{C_{SSM}}=C_{SSM}$$

It is evident that any discrete hidden state can be represented using discrete parameters and the discrete input sequence by setting the initial state $h_{-1}=0$. Therefore, the output can be calculated through a discrete convolution between the input sequence and a discrete convolutional kernel, as shown in the following Equations (9) and (10).

(9) $$\bar{K}=(\bar{C_{SSM}}\;\bar{B_{SSM}},\;\bar{C_{SSM}}\;\bar{A_{SMM}}\;\bar{B_{SSM}},\;\ldots ,\;\bar{C_{SSM}}\;{\bar{A_{SMM}}}^{L-1}\;\bar{B_{SSM}})$$

(10) $$y_{SSM}=x*\bar{K}$$

The convolutional results can be efficiently computed employing Fast Fourier Transforms (FFTs), as the convolution kernel $\bar{K}$ can be precomputed from the discrete parameters. According to the algorithm of SSMs, representative features can be extracted while accounting for sequence dependencies between inputs. This allows SSMs to replace RNNs by utilizing convolution-based computations, which are more efficient than the recurrent operations used in RNNs.

#### 3.2.2. Laplacian Positional Encoding and Random-Walk Structural Encoding

In this paper, we utilize Laplacian Positional Encoding (LapPE) and Random-Walk Structural Encoding (RWSE) to assist the spatial transformer in understanding the skeletal graph topology.

LapPE provides global positions of nodes within the graph, where nodes are considered closer if their LapPEs are more similar. LapPE is based on the eigenvectors and eigenvalues of Laplacian. The Laplacian of an undirected skeletal graph is computed using the degree matrix D and the adjacency matrix A, as presented in the following Equation (11). In this context, *L* represents the resulting graph Laplacian.

(11) L=D−A

Thereby, eigenvectors x can be obtained through solving the following Equation (12), where x are the eigenvectors and λ are the eigenvalues.

(12) Lx=λx

The calculated eigenvectors are denoted as EigenVec, having the size of $R^{N\times N_{vec}}$, and eigenvalues, EigenVal, having the size of $R^{N\times 1}$, where N is the number of nodes in the skeletal graph (16 in this paper) and $N_{vec}$ is the number of eigenvectors. Following the approaches outlined in [41], the EigenVal is expanded to match the size of $N_{vec}$, and subsequently, concatenated with EigenVec. The concatenated EigenVal and EigenVec are then processed by a multi-layer perceptron (MLP) and linearly projected to the encoding dimension, resulting in the final LapPE.

RWSE incorporates the substructures of the nodes to which the nodes belong, based on the diagonal of the m-steps random-walk matrix of the graph. This can be defined by the following Equations (13) and (14).

(13) $$RWSE=[{RW}_{ii},{RW}_{ii}^{2},\ldots ,{RW}_{ii}^{m}]\in R^{m}$$

(14) $$RW=AD^{-1}$$

The diagonal of the random-walk matrix represents the probabilities of node i returning to itself after m steps. The original RWSE process only involves a linear projection of these probabilities to the encoding dimension.

#### 3.2.3. Trajectories-Aware Temporal (TT) Module

In the TT module, the skeletal graph feature, tensor $X\in R^{T\times C\times J}$, is permuted into $X_{T}\in R^{J\times T\times C}$. All subsequent operations within the temporal module are performed on the temporal features of each joint, so we redefine the $X_{T}$ into a sequence as follows in Equation (15):

(15) $$X_{T}=\{x_{T}^{1},x_{T}^{2},\ldots ,x_{T}^{J}\}$$

where $x_{T}^{j}\in R^{T\times C}$.

##### Trajectorial Encoding

**Figure 5:**
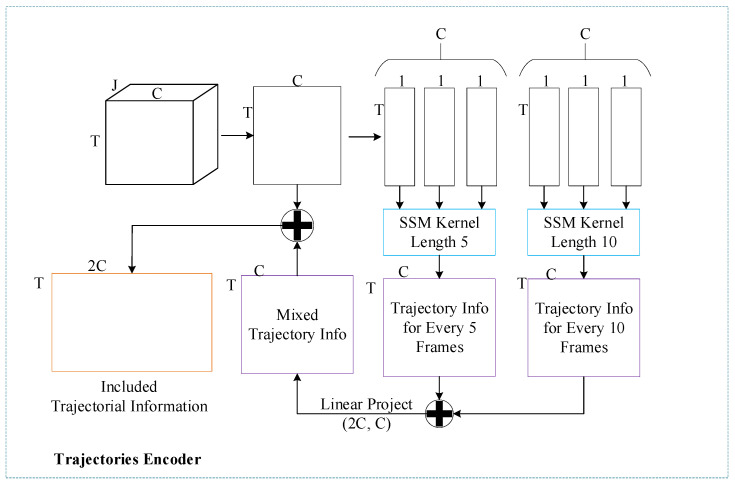
Process of trajectorial encoding.

In the TT module, a trajectory encoder is first applied to the temporal feature tensor $x_{T}$. The trajectories of the joints are sequences of coordinates with significant sequence dependencies between consecutive frames. To capture these sequence-based relationships, we employ the SSM as described in [47] to encode trajectorial information in $x_{T}$ along each dimension C. Specifically, we utilize two SSMs with kernel sizes of 5 and 10 to extract trajectorial information at short and long dependencies, resulting in ${traj}_{short}\in R^{T\times C}$ and ${traj}_{long}\in R^{T\times C}$. We then concatenate ${traj}_{short}$ and ${traj}_{long}$ and apply a linear projection to obtain a fused trajectorial encoding ${traj}_{encod}\in R^{T\times C}$. Finally, the temporal feature tensor $x_{T}$ is concatenated with ${traj}_{encod}$ to form a tensor $x_{TT}\in R^{T\times 2C}$, which contains both temporal features and trajectorial information. The entire process of trajectorial encoding is illustrated in Figure 5.

##### Temporal Transformer

**Figure 6:**
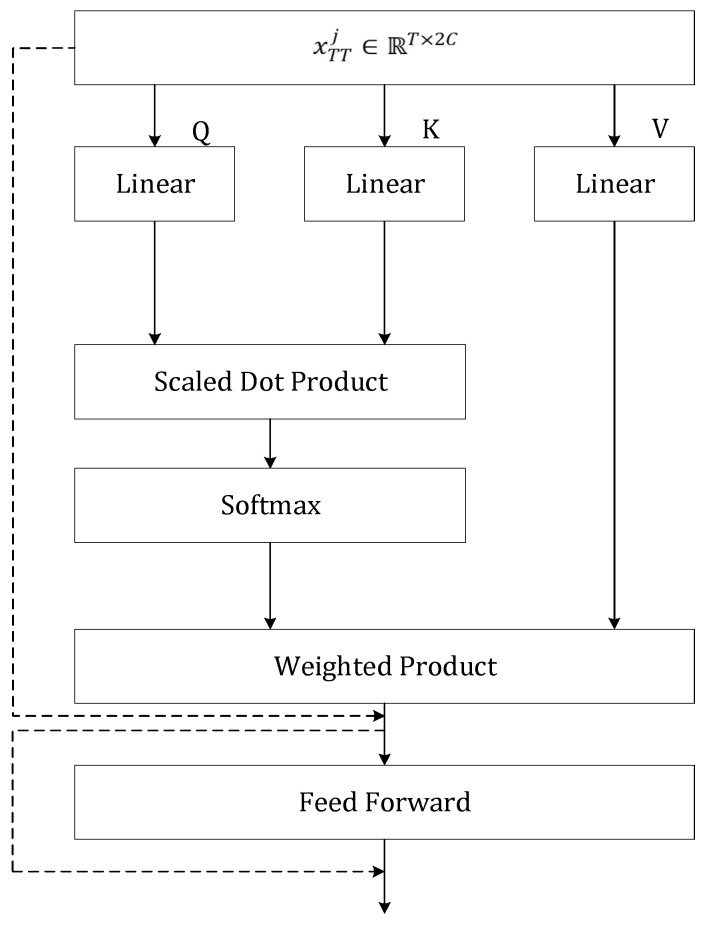
The details of the temporal transformer, with dotted arrows representing residual connections.

The architecture of the temporal transformer follows the same design principles as the basic transformer demonstrated in [24], as presented in Figure 6. Positional encoding is crucial for the transformer to aggregate features while considering the relationships between elements in the sequence. Therefore, we utilize ${traj}_{encod}$, which inherently encodes sequential dependencies, as the positional encoding for the temporal transformer. The input $x_{TT}$ is initially projected to three matrices: $Q_{T}\in R^{T\times d_{k}}$, $K_{T}\in R^{T\times d_{k}}$, and $V_{T}\in R^{T\times d_{v}}$. The global temporal relationships between frames are subsequently captured using multi-head attention, as formulated in (16). The temporal attention mechanism, denoted as ${Atten}_{T}$, is computed following the approach outlined in [24]:

(16) $${MultiHead}_{T}\left(Q_{T},\;K_{T},\;V_{T}\right)=Concat({Atten}_{T}^{1},\ldots ,{Atten}_{T}^{HeadNum})W_{T}^{O}$$

The multi-head attention result, ${MultiHead}_{T}$, is then added to the input $x_{TT}$ defined as $\bar{{MultiHead}_{T}}$. This output is then processed through a feed-forward network (${FF}_{T}$) with a residual connection, producing the temporal transformer output $y_{T}\in R^{T\times d_{v}}$. This process is described in Equation (17).

(17) $$y_{T}=\bar{{MultiHead}_{T}}+{FF}_{T}(\bar{{MultiHead}_{T}})$$

Considering all joints in the skeletal graph, the overall output of the TT module is represented as $Y^{T}=\{y_{T}^{1},y_{T}^{2},\ldots ,y_{T}^{J}\}$.

#### 3.2.4. Skeleton-Graph-Aware Spatial (SS) Module

In the SS module, the skeletal graph features $X\in R^{T\times C\times J}$ is permuted into $X_{S}\in R^{T\times J\times C}$. All subsequent operations within the spatial module are performed on the spatial graph features for each frame, so we redefine the $X_{S}$ into sequences:

(18) $$X_{S}=\{x_{S}^{1},\;x_{S}^{2},\;\ldots ,\;x_{S}^{T}\}$$

where $x_{S}^{t}\in R^{J\times C}$.

It is important to note that only the first SS module expands dimension C of $X_{S}$ from 3 to 32 through projection to enhance the capacity of the spatial module.

##### Skeletal Graph Encoder

Following the approaches described in Section 3.2.2, the LapPE and 17-step RWSE are computed initially, defined as $pos\in R^{J\times d_{e}}$ and $struct\in R^{J\times d_{e}}$, where the encoding dimension $d_{e}$ is set to 32. Since the skeletal graph topology described in Section 3.1 remains consistent across all gaits, we calculate pos and struct in advance at the beginning of the TS-ST model to avoid redundant calculation in the SS module. The precomputed pos and struct are then concatenated to form the graph encoding ${en}_{graph}\in R^{J\times {2d}_{e}}$, which is utilized in the skeletal graph encoder.

The architecture of the skeletal graph encoder is straightforward, consisting solely of a linear layer that projects ${en}_{graph}$ to match the same C dimension as $x_{s}$. The projected graph encoding is defined as ${\bar{en}}_{graph}\in R^{J\times C}$ and is subsequently concatenated with $x_{s}$ to form $x_{ss}\in R^{J\times 2C}$.

##### Spatial Transformer

**Figure 7:**
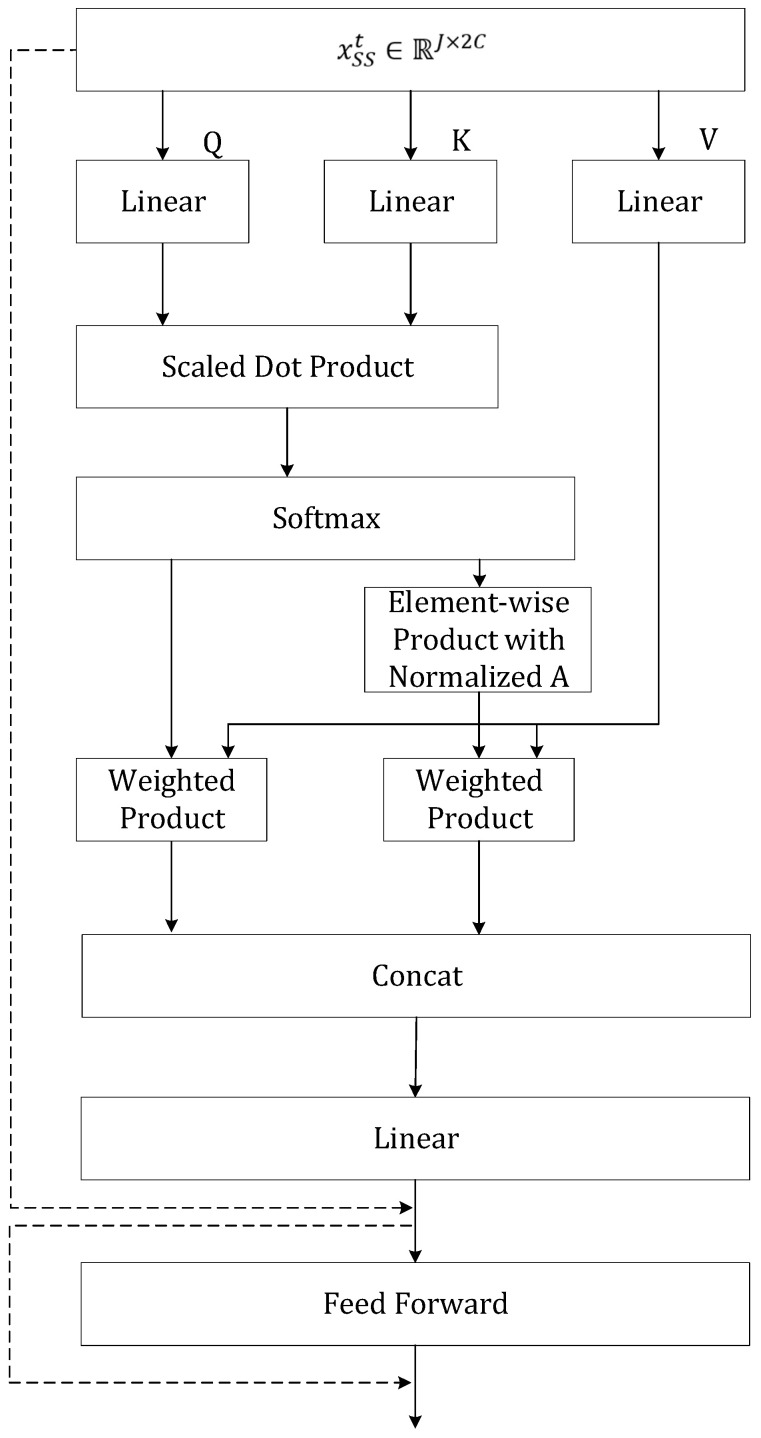
The details of the spatial transformer, with dotted arrows representing residual connections.

The architecture of the spatial transformer is similar to that of the temporal transformer, as presented in Figure 7,with enhancements made to the attention mapping to enable the model to aggregate information based on both long-range and short-range relationships.

To illustrate these enhancements, the first step involves projecting the input $x_{SS}$ to three matrices, $Q_{S}\in R^{T\times d_{k}}$, $K_{S}\in R^{J\times d_{k}}$, and $V_{S}\in R^{J\times d_{v}}$, and calculating the attention score with Equation (19).

(19) $${AttScore}_{S}=softmax(\frac{Q_{S}{K_{S}}^{T}}{\sqrt{d_{k}}})$$

As discussed previously, the attention score describes global relationships, which can also be interpreted as long-range relationships between joints in the graph. To incorporate local relationships, we follow the approach used in graph transformers by utilizing a normalized adjacency matrix $\bar{A}$. This matrix explicitly allows the attention mechanism to also account for local relationships, which can be characterized as short-range connections. This normalized adjacency matrix $\bar{A}$ can be calculated from the following Equation (20).

(20) $$\bar{A}={\hat{D}}^{-\frac{1}{2}}\hat{A}{\hat{D}}^{-\frac{1}{2}}$$

where $\hat{A}=A+I$ and $\hat{D}$ is the diagonal node degree matrix of $\hat{A}$.

We then apply an element-wise product between $\hat{A}\in R^{J\times J}$ and ${AttScore}_{S}\in R^{J\times J}$ to calculate ${AttScore}_{S}^{Graph}$, which enforces relationships only between joints that have connections in the skeletal graph:

(21) $${\bar{A}}_{weighted}={AttScore}_{S}^{Graph}=\bar{A}*{AttScore}_{S}$$

From the perspective of the graph, ${AttScore}_{S}^{Graph}$ can be interpreted as a weighted adjacency matrix. ${\bar{A}}_{weighted}$ represents the weights along the edges in the skeletal graph. As described in the algorithms presented in graph convolutional networks [48], the aggregation of information on the graph can be achieved through multiplication between the adjacency matrix and the node features, which is multiplication between our weighted adjacency matrix ${AttScore}_{S}^{Graph}$ and the projected spatial feature $V_{S}$. This is expressed as Equation (22).

(22) $${{Atten}_{S}^{short}=Agg}^{Graph}={\bar{A}}_{weighted}V_{S}={AttScore}_{S}^{Graph}V_{S}$$

Equation (22) demonstrates that the aggregation process is similar to attention mapping, with the limitation that relationships in the attention score matrix are confined to short-range connections. For long-range information aggregation, we follow the basic attention mechanism, resulting in ${Atten}_{S}^{long}$.

(23) $${Atten}_{S}^{long}={AttScore}_{S}V_{S}$$

We can then combine our global attention and local aggregation by concatenation:

(24) $${Atten}_{S}=concat({Atten}_{S}^{long},{Atten}_{S}^{short})$$

Consequently, multi-head attention for the spatial transformer is indicated in the following equation:

(25) $${MultiHead}_{S}\left(Q_{S},K_{S},V_{S}\right)=Concat\left({Atten}_{S}^{1},\ldots ,{Atten}_{S}^{HeadNum}\right)W_{S}^{O}$$

The remaining steps for the spatial transformer are identical to those of the temporal transformer.

(26) $$y_{S}=\bar{{MultiHead}_{S}}+{FF}_{S}(\bar{{MultiHead}_{s}})$$

At the end of the spatial transformer, the output is $y_{S}\in R^{J\times d_{v}}$. Considering all frames in the skeletal graph sequence, the overall output of the SS module is represented as $Y^{S}=\{y_{S}^{1},\;y_{S}^{2},\;\ldots ,\;y_{S}^{s}\}$.

#### 3.2.5. Classification Module and Loss Function

As shown in Figure 4, the TT module and the SS module operate in parallel. To fuse the output $Y^{T}\in R^{T\times d_{v}\times J}$ from the TT module and $Y^{S}\in R^{T\times d_{v}\times J}$ from the SS module, the element-wise product operation is performed between the outputs of two the modules, resulting in the fused output $Y\in R^{T\times d_{v}\times J}$. The fused output Y is then utilized as the input for both the TT module and the SS module in the subsequent TT-SS module. The TS-ST model is composed of N TT-SS modules in sequence, with the final fused output from the last TT-SS module denoted as $Y_{N}\in R^{T\times d_{v}\times J}$.

Average pooling is applied on the $R^{T\times J}$ dimensions of $Y_{N}$ to obtain the spatial–temporal representation ${\bar{Y}}_{N}\in R^{d_{v}}$. A multilayer perceptron (MLP), consisting of two fully connected layers with a SoftMax function, is used to classify emotions. The result of the SoftMax, $\hat{P}\in R^{M}$, represents the predicted probabilities of M emotions. The entire process is indicated in Equations (27) and (28).

(27) $${\bar{Y}}_{N}=AveragePooling2D(Y_{N})$$

(28) $$\hat{P}=SoftMax(MLP({\bar{Y}}_{N}))$$

The cross-entropy function is used to calculate the loss of the TS-ST model, which can be computed as follows:

(29) $$L=-Plog\hat{P}$$

where $P\in R^{M}$ is the true probabilities distribution of each emotion. As this paper focuses on single-label emotion recognition, only the probability corresponding to the true emotion is set to 1, while the probabilities for other emotions are set to 0.

#### 3.2.6. Spectral Information

Due to the parallel architecture of the TT module and the SS module, it is important to note that the SS module cannot access the temporal information among the gait sequence until the first element-wise product fusion between outputs of the TT and SS modules. Consequently, the temporal information is not incorporated in the first SS module. To address this limitation and complement the temporal information, handcrafted spectral information $x_{spectral}\in R^{J\times C}$ is concatenated with the spatial features $x_{S}^{t}\in R^{J\times C}$ at the outset of the first SS module.

To extract these spectral features, the Fast Fourier Transform (FFT) is applied to the sequence of skeletal graph features $X\in R^{T\times C\times J}$, obtaining the frequency spectrum $FS\in R^{F\times C_{f}\times J}$. The frequency spectrum represents the discrete frequency components along dimension F and the corresponding amplitudes along dimension $C_{f}$. Instead of using the entire frequency spectrum, our focus is placed on the amplitude of the low-frequency components.

**Figure 8:**
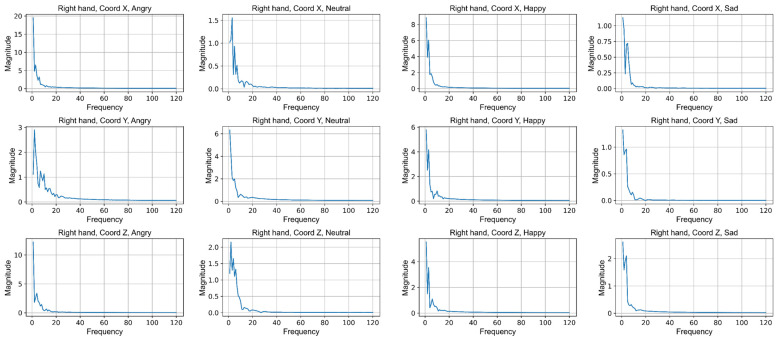
Frequency spectrums excluding zero-frequency components on coordinates *x*, *y*, and *z* of the right hand, corresponding to the expression of four different emotions. The magnitudes are expressed in the same units as the coordinates *x*, *y*, and *z*, which are determined by the camera’s view. The frequency represents the number of frequency resolutions, which is determined by the frames per second of the camera.

Figure 8 illustrates the frequency spectrums across C coordinate axes (*x*, *y*, and *z*) of the right hand, corresponding to the expression of four emotions. It is notable that the zero-frequency component has been excluded. Due to limitations in figure size and clarity, the frequency spectrums for all joints are not presented; instead, the frequency spectrums of the right hand are shown as a representative example.

As illustrated in Figure 8, high amplitudes are concentrated in the low-frequency components, highlighting the significance of low-frequency components in the gait sequence. Additionally, the peak amplitudes vary across different frequency spectra when expressing different emotions. By sorting the highest amplitudes along the *x* axis from high to low, the emotion rankings are identified as follows: anger, happiness, neutral, and sadness. Moreover, the peak amplitudes in the low-frequency components for anger and happiness are significantly higher than those for neutral and sad expressions. This suggests that the variation in amplitude within the low-frequency components can be leveraged for emotion classification.

As a result, we only use the maximum amplitude among the first 20 frequency components to represent the entire spectrum, defining it as $x_{spectral}\in R^{J\times C}$, where J and C retain the same values as in the original skeletal graph features, specifically, 16 and 3. In summary, the spectral features not only complement the temporal information overlooked by the TT module but also serve as affective features that enhance emotion classification.

### 3.3. Emotional Gait Response for Robot

In our research, we used the NAO robot to realize the predefined emotional gait response. The robot stands at a height of 57.4 cm and weighs 5.4 kg. It features a total of 25 degrees of freedom (DOF), with 2 DOF in the head, 5 DOF in each arm, 1 DOF in each hand, and 6 DOF in each leg. These DOF provide a broad range of motion, enabling natural movements. Additionally, the NAO robot is equipped with cameras, ultrasonic sensors, touch sensors, and an inertial unit for environmental perception. For interactions withhumans, microphones and speakers are placed in the robot.

**Figure 9:**
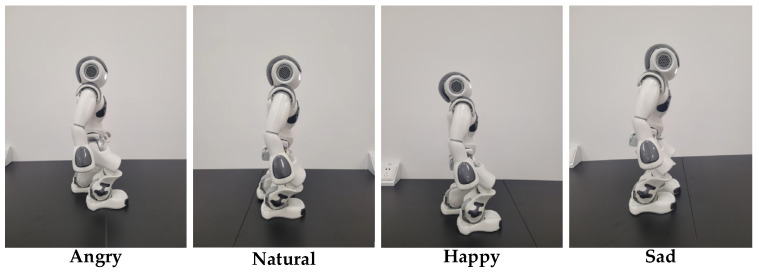
Emotional-gait response of NAO with four emotions.

To enable the NAO robot to express emotions through gait, we have defined four walking patterns corresponding to the observed human emotions, as presented in Figure 9. When expressing anger, the NAO robot lowers its head slightly and walks at the fastest speed, taking the largest steps among the four emotional responses. To convey a neutral emotion, the NAO walks at a medium speed without bending its torso or lowering its head. In contrast, to express happiness, the NAO raises its head, bends its back, and walks at a high speed with a large step length. To express sadness, the NAO lowers its head significantly, bends its torso forward, and walks at the lowest speed with the smallest step length. The detailed parameters for these four emotional expressions are presented in Table 1.

## 4. Experiments and Results

### 4.1. Emotion-Gait Dataset and Robot Platform

In this paper, we utilize the Emotion-Gait dataset provided by [18], which consists of 2177 3D emotional gait sequences categorized into four emotions, angry, neutral, happy, and sad. The dataset includes 1835 gaits from the Edinburgh Locomotion Mocap Database (ELMD) and an additional 342 sequences collected by the authors of [18]. The skeletal graphs in the Emotion-Gait dataset consist of 16 joints, and the maximum sequence length is 240 frames.

We utilize the NAO robot, supported by Softbank Robotics China (Shanghai, China), for our research. The robot interactions are managed through the NAOqi operating system, developed by Softbank Robotics.

### 4.2. Implementation Details and Training Configurations

Our TS-ST model is composed of two TT-SS modules. The transformer architecture in both the TT and SS modules follows similar settings. In the first module, both the dimension $\;d_{k}$ and the dimension $d_{v}$ are set to 32, while in the second module, $d_{k}$ and $d_{v}$ are set to 32 and 64, respectively. For all transformers, the output projection dimension is consistent with the dimension $d_{v}$. Additionally, in the two fully connected layers within MLP, the input and output dimensions are 64 and 32 and 32 and 4, respectively.

In accordance with the method outlined in [18], the only preprocessing step involves transforming all gaits to match the viewpoint of the first gait in the dataset. The preprocessed dataset is then split into training and test sets with a 9:1 ratio and batched into sets of eight.

We train the model using an NVIDIA RTX 4080 GPU (Dell (China) Co., Ltd., Xiamen, China), implemented in the PyTorch 2.3.1 framework. The training optimizer is RMSprop with a learning rate of 1E-4, momentum of 0.5, and weight decay of $1\times 10^{-4}$. The training runs for 200 epochs, and we apply learning rate annealing with a decay ratio of 0.5 every 50 epochs after the initial 75 epochs.

The TS-ST model contains 0.14 M parameters, and its computational cost, measured in FLOPs, is 3.70 G when using a batch size of eight. Due to the limitations imposed by the Python version supported by the NAOqi operating system and the computational power of the NAO robot, emotion prediction operations are performed on an external computer. The computer receives gait image sequences captured by the NAO robot, processes them, and sends the predicted results back to the robot.

### 4.3. Comparison with State-of-the-Art Approaches

We utilize accuracy as the metric to evaluate the performance of our model on the Emotion-Gait dataset. The accuracy is defined as Equation (30), where TP and TN are the numbers of true positive samples and true negative samples, respectively, and Ts is the total number of samples in the test set.

(30) $$Accuracy=\frac{TP+TN}{TS}$$

The results are presented in Table 2, where we compare our TS-ST model with state-of-the-art gait-based emotion classification methods. ST-GCN [17] employs spatial graph convolutional networks to extract graph-aware spatial features from human skeleton data and utilizes convolutional neural networks to capture temporal features. In contrast, STEP [18] reduces the number of layers in the ST-GCN architecture, demonstrating improved performance in gait-based emotion recognition. Both TT-GCN and G-GCSN are variants of the ST-GCN framework. TT-GCN [36] introduces casual temporal convolution networks, capturing dependencies between steps in the gait sequence, while G-GCSN [49] incorporates global links in spatial graph convolutional neural networks to capture spatial features with the global context. These approaches are categorized as graph-based methods, as summarized in Table 3. TNTC [37] encodes the gait sequence into two image streams processed by ResNet and then fuses the features from both streams using a transformer model. Similarly, ProxEmo [5], encodes the gait sequence into images, from which emotion representations are extracted using group convolutional networks.

The results from other gait-based emotion classification methods are extracted from the original papers and evaluated using the Emotion-Gait dataset, with a train-test split ratio of 9:1. The methods are classified into three categories: robot-based, graph-based, and transformer-based approaches, as indicated in the table. It is evident that our TS-ST model outperforms other graph-based and transformer-based methods in terms of accuracy, achieving notably better performance than the most advanced robot-based approach for gait emotion recognition.

### 4.4. Performance Analysis

To further evaluate the performance of our TS-ST model across each emotion, we utilize precision, recall, and F1-score as evaluation metrics. These metrics are computed using the following equations.

(31) $$Precision=\frac{TP}{TP+FP}$$

(32) $$Recall=\frac{TP}{TP+FN}$$

(33) $$F1=2\cdot \frac{Precision\cdot Recall}{Precision+Recall}$$

The results are presented in Table 4, where our TS-ST model demonstrates strong performance in classifying the emotion of anger, achieving both high precision and recall, which results in a high F1-score of 0.9610. Based on the F1-scores, our TS-ST model also performs effectively in classifying the emotions of neutrality and happiness, with scores of 0.8000 and 0.8182, respectively. However, there is a noticeable disparity between the precision and recall for the classification of neutrality, suggesting frequent misclassification of other emotions as neutral. In contrast, the model’s performance in predicting sad emotions is relatively poor, with a low F1-score of 0.5625, indicating a less accurate classification compared to other emotions.

We also evaluate the performance of the TS-ST model with varying numbers of TT-SS (Trajectories-Aware Temporal and Skeleton-Graph-Aware Spatial Module in parallel) modules under the metric of accuracy. In this evaluation, the number of the TT-SS module with 32 $d_{k}$ and 32 $d_{v}$ is modified. The results are shown in Table 5 with implementation details of $d_{k}$ and $d_{v}$ in each TT-SS module’s transformer. The results indicate that the TS-ST model with two TT-SS modules yields the highest performance. The accuracy of the model with a single TT-SS module is slightly lower (approximately 0.5%) compared to the two-module configuration, suggesting that affective representations are not fully captured with just one TT-SS module. In contrast, the performance of the model with more than two TT-SS modules shows a significant decline, indicating overfitting to the training data and incorrect extraction of affective representations.

Additionally, the performance of the TS-ST model, which integrates spectral information with the Trajectories-Aware Temporal (TT) module and the Skeleton-Graph-Aware Spatial (SS) module, is evaluated. The spectral information is concatenated with the input to the first SS module, following the methodology described in Section 3.2.6. The accuracy results are presented in Table 6, where concatenating spectral information with the input of the first SS module presents the highest performance, achieving an accuracy of 84.15%. In contrast, concatenating spectral information with the input to the TT module results in significantly lower performance, with an accuracy of 77.91%. This decrease in performance can be attributed to the distortion of local temporal information caused by the inclusion of spectral information during trajectory encoding, which primarily focuses on local temporal features. The distortion also affects the model’s performance when spectral information is concatenated with the inputs of both the TT and SS modules, with an accuracy of 83.67%.

### 4.5. Ablation Study

To verify the effectiveness of our trajectorial encoding (TE), graph encoding (GE), and spectral information, we train the model with only one type of encoding and without spectral information, evaluating the performance on the test data using accuracy and average precision (AP) metrics. The average precision is calculated from Equation (34), where N is the number of thresholds, $R_{n}$ is the recall at the $n_{th}$ threshold, and $P_{n}$ is the precision at the $n_{th}$.

(34) $$AP=\sum_{n}^{N}(R_{n}-R_{n-1})\times P_{n}$$

In the actual calculation, the thresholds are determined dynamically based on the data and are linearly spaced to N values. The precision–recall curves are generated to obtain precisions and recalls at different thresholds.

We compared the performances of different models with the base model, which only includes the spatial transformer and the temporal transformer described in the methodology section, and excludes the TE, GE and spectral information. The results are presented in Table 7, with the Mean Average Precision (MAP) being the mean of the average precision of each class.

The results show that the accuracy of models using any type of encoding exceeds that of the base model by more than 3%, indicating that TE and GE contribute significantly to capturing temporal and spatial features in the skeletal graph sequences, respectively. This verifies the significance of sequence dependencies between frames, as well as the positional and structural information within the graph. According to the average precision results, TE improves performance in predicting neutral and angry emotions, while GE significantly enhances performance in predicting sad emotions. Furthermore, the model with both TE and GE shows better performance compared to models using either encoding individually, demonstrating the complementary effect between TE and GE, especially for the classification of sad emotion, which is evident from the improvement in average precision. Finally, there is a clear improvement in the model with spectral information, as the average precision for each emotion increases significantly compared to the base model. This is because spectral information provides global temporal information to the SS module, addressing the lack of temporal representations in the early stages of the spatial stream. This allows our spatial model to aggregate features from the joints on the same skeletal graph while being aware of the differences in joints in the temporal domain in the initial spatial module.

## 5. Conclusions and Future Works

In this paper, we present a new Gait-to-Gait Emotional HRI system, implemented on the NAO robot, to address the gap between human gait-based emotion recognition and robot emotional-gait response, applying it to the NAO robot. To overcome challenges in capturing both temporal and spatial information, we propose a new gait-emotion classification model, TS-ST, which can effectively extract sequence dependencies across frames and encode positional and structural information from skeletal graphs, by incorporating the space state model and the graph transformer. Our TS-ST can recognize four human emotions: anger, neutrality, happiness, and sadness. Moreover, the NAO robot is capable of walking with corresponding preset emotional gait responses to predicted human emotions, completing an emotional interaction. The evaluation of the Emotion-Gait dataset shows that our TS-ST model outperforms the state-of-the-art robot-based gait-emotion classification model.

In future work, we plan to integrate the affective features of gaits alongside joint coordinates to enhance our model’s performance. Specifically, we will use regression prediction to constrain the representation of gait emotion with these affective features. Additionally, we will explore efficient methods for extracting temporal representations to significantly reduce the computational cost of our model. Our current research is limited to the Emotion-Gait dataset. Consequently, we will investigate the generalizability of the model on other gait-emotion datasets.

## Figures and Tables

**Table 1:** Emotional gait parameter settings for NAO.

Emotion	Head Degree [deg]	Torso Degree [deg]	Step Speed [step/s]	Step Length [mm]
Angry	10	0	1.0	80
Natural	0	0	0.5	40
Happy	−10	−5	0.8	60
Sad	20	10	0.2	20

**Table 2:** Comparison of our TS-ST with state-of-the-art methods on accuracy with Emotion-Gait dataset.

Method	Accuracy (%)
ST-GCN [17]	65.62
STEP [18]	78.24
TNTC [37]	79.52
TT-GCN [36]	80.11
G-GCSN [49]	81.5
ProxEmo [5]	82.4
TS-ST (Ours)	**84.15**

The highest accuracy is highlighted in bold.

**Table 3:** Classification of state-of-the-art methods.

	Robot Based	Graph Based	Transformer Based
ST-GCN [17]	×	√	×
STEP [18]	×	√	×
TNTC [37]	×	×	√
TT-GCN [36]	×	√	×
G-GCSN [49]	×	√	×
ProxEmo [5]	√	×	×
TS-ST (Ours)	√	√	√

**Table 4:** Performance of TS-ST model on each emotion.

	Precision	Recall	F1-Score
Angry	0.9652	0.9569	0.9610
Natural	0.7368	0.8750	0.8000
Happy	0.8182	0.8182	0.8182
Sad	0.7500	0.4500	0.5625

**Table 5:** Performance of TS-ST model with different numbers of TT-SS modules.

Number of TT-SS Modules	1st Module [$d_{k},d_{v}$]	2nd Module [$d_{k},d_{v}$]	3rd Module [$d_{k},d_{v}$]	4th Module [$d_{k},d_{v}$]	Accuracy (%)
1	[32, 64]				83.73
2	[32]	[32, 64]			**84.15**
3	[32]	[32]	[32, 64]		81.43
4	[32]	[32]	[32]	[32, 64]	79.39

The highest accuracy is highlighted in bold.

**Table 6:** Performance evaluation of the TS-ST model with spectral information integrated into the TT and SS modules.

Modules with Spectral Info	Accuracy (%)
TT module with Spectral Info	77.91
SS module with Spectral Info (Original TS-ST)	**84.15**
TT module and SS module with Spectral Info	83.67

The highest accuracy is highlighted in bold.

**Table 7:** Ablation analysis of TS-ST.

Method	Angry (AP)	Natural (AP)	Happy (AP)	Sad (AP)	MAP	Accuracy (%)
TS-ST without GE, TE and Spectral Info	0.9001	0.6386	0.3997	0.1372	0.5189	78.88
TS-ST without GE and Spectral Info	0.9307	0.7002	0.4951	0.1465	0.5686	82.15
TS-ST without TE and Spectral Info	0.9387	0.6872	0.4545	0.2742	0.5886	82.34
TS-ST without Spectral Info	0.9187	0.6750	0.4919	0.4540	0.6349	83.20
TS-ST	**0.9589**	**0.7684**	**0.7504**	**0.5659**	**0.7609**	**84.15**

The highest accuracy and average precisions of each emotion are highlighted in bold.

## Data Availability

The data presented in this study are openly available in (https://go.umd.edu/emotion-gait) accessed from 28 October 2019.
